# Acute Diverticulitis as an Atypical Presentation of Alpha-Gal Syndrome

**DOI:** 10.7759/cureus.111671

**Published:** 2026-06-28

**Authors:** Emaan Salam, VonPatrick Soliman DelaRosa, Manish Trivedi

**Affiliations:** 1 Internal Medicine, AtlantiCare Regional Medical Center, Atlantic City, USA; 2 Infectious Disease, AtlantiCare Regional Medical Center, Atlantic City, USA

**Keywords:** abdominal abcess, alpha-gal syndrome, complicated diverticulitis, lone star tick, lower abdominal pain

## Abstract

Alpha-gal syndrome (AGS) is an immunoglobulin E (IgE) mediated hypersensitivity reaction caused by tick bites. The body's immune response to tick bites can lead to the production of anti-alpha-gal IgE antibodies, disrupting the normal oral tolerance to food allergens. AGS typically results in delayed anaphylaxis after consuming red meat or certain medications and immediate anaphylaxis following tick bites; however, isolated gastrointestinal manifestations are increasingly being reported.

We present a case of a 38-year-old female patient with a history of AGS who developed acute sigmoid diverticulitis with associated pelvic abscess, likely related to her AGS. Despite initiation of broad-spectrum intravenous antibiotics, the patient experienced persistent severe abdominal pain requiring diagnostic laparoscopy; however, bowel resection was ultimately avoided. The patient improved with continued intravenous antibiotics and had complete symptom resolution on follow-up. This case highlights the expanding gastrointestinal spectrum of AGS and raises the concern of an association between AGS-related immune dysregulation and inflammatory colonic pathology such as diverticulitis. Recognition of atypical gastrointestinal presentations of AGS is essential to reduce delays in diagnosis and misclassification as other gastrointestinal disorders.

## Introduction

Alpha-gal syndrome (AGS) is an immunoglobulin E (IgE) mediated hypersensitivity reaction caused by tick bites, particularly the lone star tick (*Amblyomma americanum*), specifically reacting to a carbohydrate found in tick saliva and noncatarrhine mammal tissues [1]. The body's immune response to tick bites can lead to the production of anti-alpha-gal IgE antibodies, disrupting the normal oral tolerance to food allergens. AGS typically results in delayed anaphylaxis after consuming red meat or certain medications and immediate anaphylaxis following tick bites. AGS has many novel features that broaden the paradigm of food allergy, including that reactions are delayed three-six hours after exposure and patients have frequently tolerated red meat for many years prior to the development of allergic reactions [2]. Many patients present with vomiting, diarrhea, and abdominal pain and carry a diagnosis of chronic diarrhea, irritable bowel syndrome or gastrointestinal food allergy syndrome prior to AGS diagnosis and it is important to create a resource for identifying and managing this unique allergic syndrome [2].

## Case presentation

**Figure 1:**
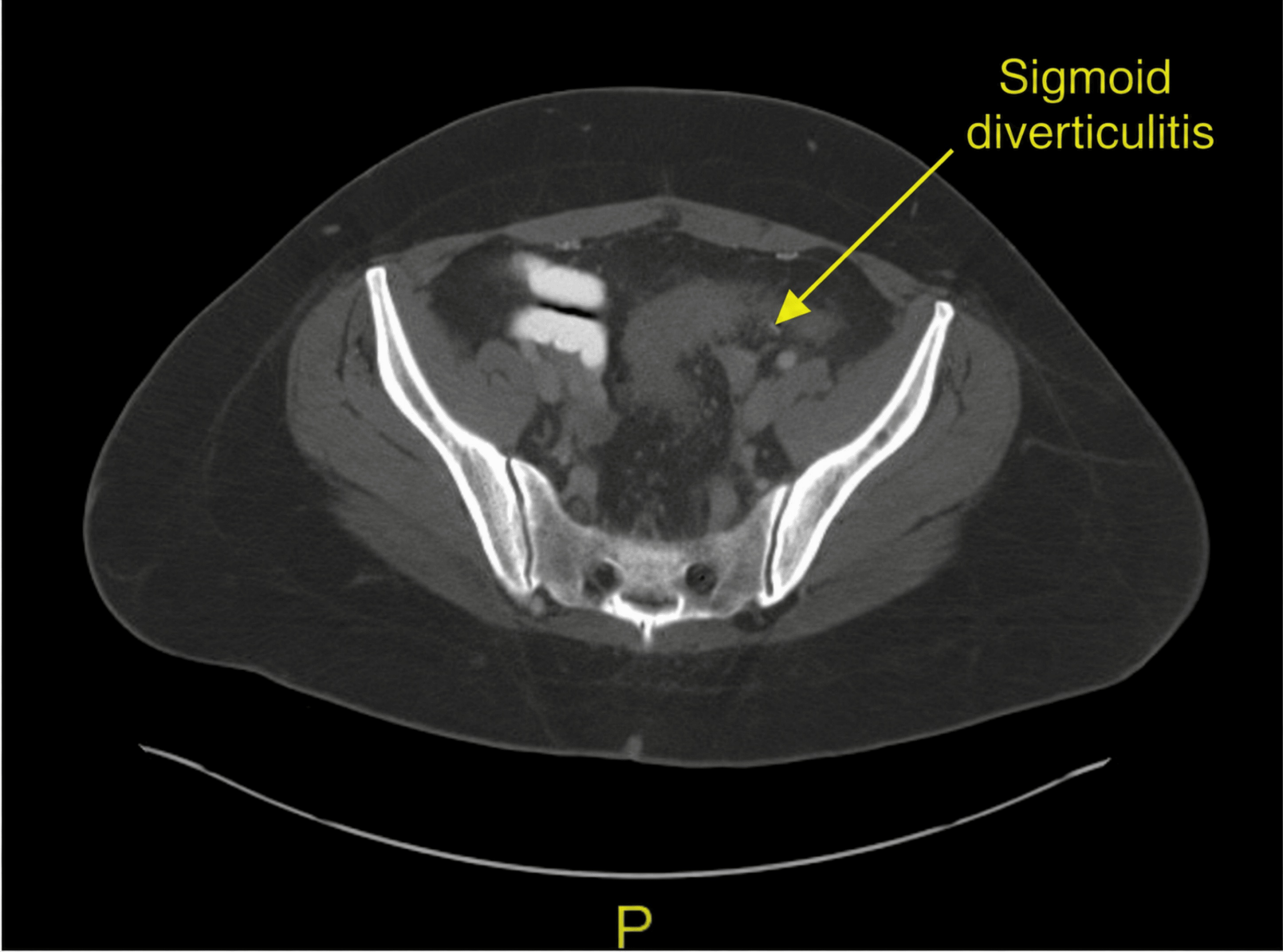
Sigmoid diverticulitis with collection of fluid compatible with developing abscess

Our patient is a 38-year-old woman presenting with worsening abdominal pain and rectal bleeding. Her past medical history includes AGS following a lone star tick bite in 2020. Within the past year she had an episode of infectious colitis and a hospitalization due to acute diverticulitis complicated by micro-perforation. The patient's diet primarily consisted of avoiding pork, dairy, and red meat due to her diagnosis of AGS. Physical exam was significant for hyperactive bowel sounds with tenderness in the lower quadrants. CT scan of the abdomen revealed an acute inflammatory process in the sigmoid colon indicative of diverticulitis with a fluid collection in the left hemi-pelvis suggestive of a developing abscess (Figure 1). She was admitted and started on intravenous antibiotics. However, the patient's symptoms did not improve over the next few days and she continued to have severe abdominal pain. Subsequently she was taken to the operating theater for laparoscopic washout and possible resection if warranted. Laparoscopy revealed that the sigmoid colon was thickened and acutely inflamed without evidence of fecal drainage. There was a loop of small bowel with omentum adherent to one small aspect of the sigmoid colon.The surgical team decided that a resection and colostomy were not warranted. Patient was continued on IV antibiotics and her symptoms improved over the course of the next few days. She was eventually discharged on IV ertapenem for more more week. Patient was then seen in the outpatient setting and her symptoms had resolved.

This case was previously presented as a poster in the Medical Education Research Day 2024 at AtlantiCare on April 23, 2024.

## Discussion

AGS typically presents with urticaria and other anaphylactic features, but isolated gastrointestinal symptoms are increasingly reported. The association between AGS and the IgE response in the colon leading to inflammation needs to be studies further. In 3-20% of AGS cases, patients report isolated gastrointestinal symptoms without anaphylaxis [2]. Misdiagnosis as irritable bowel syndrome often occurs due to lack of awareness of AGS presenting as isolated gastrointestinal symptoms [3]. Up to 3% of the population in the Southeastern United States are considered to have clinical AGS [4], but many cases remain undiagnosed or misdiagnosed as other illnesses [5]. A high suspicion for AGS is crucial especially in areas with prevalent lone star ticks to reduce the risk of misdiagnosis when patients present with gastrointestinal symptoms. 

## Conclusions

This case report underscores the importance of recognizing and managing AGS in patients presenting with acute conditions such as diverticulitis, beyond the the typical symptoms of urticaria and anaphylaxis. Raising awareness about this syndrome and its various clinical presentations can lead to a reduction in misdiagnoses. Further research is needed to better understand the pathophysiology of AGS, including mast cell infiltration in colonic tissue of ABS patients with diverticulitis.
